# Synthesis, Spectroscopic and Thermal Characterization of Copper(II) and Iron(III) Complexes of Folic Acid and Their Absorption Efficiency in the Blood

**DOI:** 10.1155/2009/979680

**Published:** 2009-09-06

**Authors:** E. Hamed, M. S. Attia, K. Bassiouny

**Affiliations:** ^1^Department of Chemistry, Faculty of Science, Ain Shams University, Cairo, Egypt; ^2^Department of Biochemistry, Genetic Engineering and Biotechnology Research Institute (GEBRI), Menoufia University, Sadat City, Egypt

## Abstract

The absorption efficiency of any drug in blood is of prime importance. Compounds having the general formula: K_n_[M(FO)_2_(H_2_O)_2_] · *x*H_2_O, where (M = Cu(II) or Fe(III), n = 2 or 1, FO = folate anion, *x* = 2 or 3 with respect), were prepared, and their absorption efficiency in rodent's blood was determined. The obtained compounds were characterized by elemental analysis, infrared as well as thermogravimetric analysis and polarization of light. The results suggest that the two folate complexes were formed in 1 : 2 molar ratio (metal : folic acid) which acted as a bidentate ligand through both carboxylic groups. Polarization of light proved that the folate complexes have symmetric geometry. Biological application proved that Cu(II) and Fe(III) complexes were absorbed more efficiently in rodent blood than folic acid itself.

## 1. Introduction

**Figure 1:**
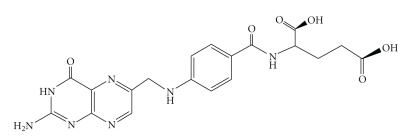
Structure of folic acid.

Folic acid as reported in the figure, N-(4{[(2-amino-4-oxo-1, 4-dihydropteridin-6-yl)methyl]amino}benzoyl)-L-glutamic acid is from a group of vitamins called water soluble vitamins which contain B-Group vitamins and the other group. It is fat-insoluble vitamins, acting as a coenzyme in many single carbon transfer reactions in the synthesis of DNA, RNA, and protein components [1]. It is called pteroyl-L-glutamic acid (PGA), see Figure 1. The occurrence of folic acid in nature is not in appreciable amounts, though it is assimilated in the body and is converted to the active cofactor form of the vitamin [2]. Folic acid deficiency results in DNA strand breaks [3], DNA hypomethylation [4], and abnormal gene expression [5]. 

It has been shown that folic acid supplementation can significantly reduce the risk of these disorders [6–14]. Folates are the key cofactors in one-carbon metabolism. Together with other substrates and cofactors it is involved in the synthesis of the purine ring, conversion of 2-deoxyuridine monophosphate to thymidine monophosphate, via *S*-adenosylmethionine. Over 80 methylation reactions are known to date [15, 16]. Given the importance of one-carbon metabolism in a broad variety of metabolic pathways, it is not entirely surprising that abnormal, notably low, folate status is associated with risk for seemingly unrelated developmental anomalies and diseases, ranging from neural tube defects, Alzheimer dementia, pregnancy complications, inflammatory disease, osteoporosis and cancer to coronary artery disease. Severe micronutrient deficiencies usually cause readily recognizable symptoms, such as anemia in folate deficiency, but the effects of subclinical deficiencies on disease development are not readily apparent, since they usually operate over prolonged periods of time [17]. The structural diversity encountered in metal-folate complexes could be attributed to the versatile ligational behavior of the carboxylate group which can function like a bidentate ligand binding to a single metal or alternatively as a bridging bidentate ligand coordinating to two metals or as a monodentate ligand [18, 19]. The three different coordination modes were reported in literature [20, 21]. The aim of the present work is to demonstrate the enhancement of the absorption efficiency of folic acid in blood by using biological saving metals as Fe and Cu which already exist in human blood and also to show the way in which these transition metals combine with folic acid. The composition and structure of the complexes were identified using: elemental analysis (C,H,N), FTIR, atomic absorption, light polarization, and TGA techniques. 

## 2. Experimental: Materials and Instrumentation

### 2.1. Materials

All chemicals used in the present study are reagent grade,

1. Folic acid, Adwic (C_19_H_19_N_7_O_6_ · 2H_2_O) (Figure 1) used without further purification.
2. The following metal basic carbonates were used in preparing the solid folate complexes after determining their metal content after dissolution in the least amount of dilute nitric acid, by atomic absorption spectrometry
  - CuCO_3_ · Cu(OH)_2_ · H_2_O
  - Fe_2_(CO_3_)_3_ · 3Fe(OH)_3_ · 5H_2_O.

### 2.2. Instrumentation

**Elemental Analysis**

C, H, and N were determined at the microanalytical laboratory, Cairo and Ain Shams Universities using a Perkin Elmer 2400 CHN elemental analyzer. Copper and iron were determined by atomic absorption spectrometry (AAS), using Perkin Elmer AAS 3100. A given weight of the complex was heated with concentrated nitric acid nearly to dryness. The resulting metal nitrate was dissolved in distilled water and the solution made to volume in a measuring flask. Standard metal solution series is prepared by diluting calculated volumes of a standard stock solution of the metal to the mark in a volumetric flask. The hollow cathode lamp of our metals concerned are used, the current adjusted to the recommended value and the metal line. (*λ* nm) is selected to give the maximum absorption using the appropriate monochromator slit width. The absorbance of the sample is read and the concentration of the metal is determined from the calibration curve constructed from the absorbances of the standard solutions.

**Atomic Absorption Measurements**

Thermogravimetric analysis was carried out using a Perkin-Elmer 7 Series thermal analyzer. The measurements were carried out under nitrogen atmosphere at a heating rate 10°C/min.

**Optical Measurements**

Polarimetric measurement was recorded on a Courtesy Messrs, Hilgher and Watts, Ltd., London polarimeter. The field of view appears dark (which occurs when the axes of the two prisms are at right angles to each other).

**I.R. Spectra**

I.R. spectra of the solid complexes were recorded on a Perkin-Elmer spectrometer Model Jasco FTIR-300E Fourier Transform Infrared Spectrometer, using KBr discs in the range 400–4000 cm^−1^.

**Solubility**

The solubility of the prepared complexes was determined by shaking few milligrams of the complex with about 25 mL of aliquots of distilled water in a water thermostat at 25°C ±0.2 for about 3 hours. The suspension was rapidly filtered and measured aliquots of the filtrate titrated against standard HCl solution using methyl orange as indicator (Table 1).

**Synthesis of Metal Complexes**

*Cu-folates,* *K*_2_[*Cu*(*FO*)_2_(*H*_2_ *O*)_2_] · 2*H*_2_ *O* *(the complex color is yellowish green).* The complex was prepared by mixing (0.1 mol) of the metal carbonate with 0.2 mol of folic acid in ∼50 mL of distilled water. (The pH of the folic acid solution is adjusted to about 7.6–7.8 by adding 0.2 M of KHCO_3_ solution and warmed at about ∼60°C before adding the metal carbonate). After complete reaction, the solution was concentrated to about 25 mL, it was then cooled in ice-cold water where crystals of copper folate complex separated out on addition of little ethyl alcohol, filtered and recrystallized from warming water, washed with ethyl alcohol and kept in a vacuum desiccators over dried silica gel. *Fe-Folates,* *K*[*Fe*(*FO*)_2_(*H*_2_ *O*)_2_] · 3*H*_2_ *O* *(the complex color is dark brown).* A similar procedure as that described for copper folate complex was carried out, for the preparation of the 1 : 2 iron complex.

**Ethical Considerations**

All animal experimental procedures had full approval from the Animal Ethics Committee of Menoufia University.

**Animals**

White albino mice were obtained from the experimental animal house of the Genetic Engineering & Biotechnology Institute, Menoufia University.

All mice had free access to commercial pelleted food and tap water. The mice were maintained at an ambient temperature of 22°C with 12 hours light/dark cycles.

**Drug Administration Procedure**

A group of male mice was given orally 100 mg/kg body weight of folic acid or its complexes (copper-folate and iron-folate), all preparations are dissolved and suspended in distilled water, five individuals were used for each treatment of folic acid and its complexes. Two hours after treatment mice were killed and plasma collected for investigations.

## 3. Results and Discussion

Results of elemental analysis and some physical characteristics of the obtained complexes are shown in Table 1. The complexes are air stable, and they have high-melting point. Their solubility in water is ~ (10^−1^ mol) in H_2_O. Elemental analysis data of the complexes indicate that the complexes have the 1 : 2 stoichiometry (metal : ligand) with general formulae shown in (Table 1). 

### 3.1. Optical Measurements

The polarimeter consists of a monochromatic light source, a polarizer, a sample cell, a second polarizer, which is called the analyzer, and a light detector. The analyzer is oriented 90° to the polarizer so that no light reaches the detector. When an optically active substance is present in the beam, it rotates the polarization of the light reaching the analyzer so that there is a component that reaches the detector. The angle that the analyzer must be rotated to return to the minimum detector “*α*” signal is the optical rotation angle.

**Figure 2:**
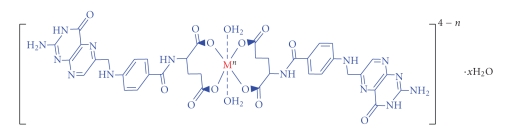
The expected structure of the two complexes.

Polarimetric measurements on solutions of the two complexes showed that both complexes possess no angle of rotation (*α* = 0), that is, having plane of symmetry. It is thus concluded that they have an octahedral structure with two trans coordinate water molecules, as shown in Figure 2.

### 3.2. Infrared Spectra

The essential infrared data are summarized in Table 2. Folic acid exhibits a very strong absorption band at 1718 cm^−1^ due to the stretching vibration of *ν*(C=O) of free ketonic of the carboxylic group [21]. This group is shifted or disappeared in the spectra of its complexes accompanied by the appearance of two bands in the 1569–1631 cm^−1^ range due to *ν*_as_(COO−) and one in the 1350–1413 cm^−1^ range assigned to *ν*_s_(COO−). Accordingly, the antisymmetric and symmetric stretching vibration modes (*ν*_as_(COO−) and *ν*_s_(COO−)) of the COO− group should help in elucidating the structure of our complexes [22].

The direction of the frequency shift of the *ν*_as_(COO−) and the *ν*_s_(COO−) bands with respect to those of the free ion depends on the coordination mode of the COO− group with the metal ion. Nakamoto and McCarthy [21, 23] claimed that if the coordination is monodentate the *ν*_as_(COO−) and *ν*_s_(COO−) will be shifted to higher and lower frequencies, respectively. Whereas, if the coordination is chelating bidentate or bridging bidentate both *ν*_as_(COO^−^) and *ν*_s_(COO^−^) frequencies will change in the same direction. This is because the bond orders of both C=O bonds would change by the same amount. Based on these facts and comparing the *ν*_as_(COO^−^) and *ν*_s_(COO^−^) frequencies of the folate complexes by the *ν*_as_(COO^−^) and *ν*_s_(COO^−^) frequencies of potassium carboxylate [24], as shown in Table 3 and Figure 2 one can say that all the prepared complexes are metal chelats in contradistinction to what is described in [21], because both *ν*_as_(COO^−^) and *ν*_s_(COO^−^) frequencies will change in the opposite direction this indicates that the carboxylic group is monodentate coordinate. The broad stretching vibration of OH^−^ group *ν*(O–H) occurred as expected [25] at ~3397 cm^−1^. The amide fragments are shown in (Table 2) by i.r. about 3200 cm^−1^ (*ν* NH), in 1652–1658 cm^−1^ region (*ν*(C=O) amide I), and about 1520 cm^−1^ (*δ*(NH) amide II) [26–28]. 

### 3.3. Thermogravimetric Analysis

**Figure 4:**
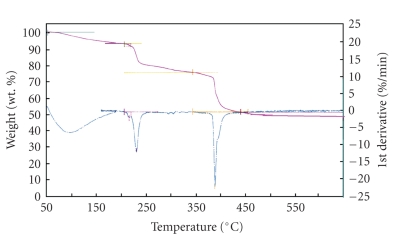
TG and DTG of copper complex.

Thermal analysis curves (TG/DTG) of folic acid and its transition metal complexes are studied and interpreted as follows. The folic acid ligand melts at 220°C with simultaneous decomposition [20, 21]. From the TG curve for copper folate complex (Figure 4), it appears that the sample decomposes in four endothermic peaks over the temperature range (0–600°C). The first step occurs from 50–170°C corresponding to the loss of four H_2_O molecules representing weight loss (obs. = 6.66% and calc. = 6.61%). The second and third steps from 170°C–599°C corresponding to the loss of C_7_H_32_N_14_O_11_(organic part) molecules, representing weight loss (obs. = 44.13% and calc. = 44.79%). The last one over 600°C was accompanied by mass loss (obs. = 49.21% and calc. = 48.60%) as metallic and carbon residue are the final product.

The thermal decomposition of iron folate complex, occurs completely in four steps. The first step in the range 50.7°C–192.4°C corresponding to the loss of 5 H_2_O molecules representing weight loss (obs. = 8.92% and calc. = 8.48%). The second and third steps from 218°C–596°C corresponding to the loss of C_7_H_31_N_14_O_11_(organic part) molecules representing weight loss (obs. = 46.3% and calc. = 45.9%). The last one over 600°C was accompanied by mass loss (obs. = 44.74% and calc. = 44.77%) as metallic and carbon residue is the final product. 

### 3.4. Biological Applications

Atomic absorption data (Tables 4  and 5) proved that the values for the concentration of metal ion in blood in the presence of the folic acid are higher than those in absence of the folic acid. This means that folic acid withdraws the metal ion from the body forming the complex in blood. It may be concluded that the folic acid in the complexed form absorbed more efficienty in the rodent blood than the folic acid itself. 

Inspection of the results shown in Table 4 reveals that the copper and iron contents in mice blood increases significantly on feeding the rodents with folic acid copper and iron complexes. The absorption of folic acid complexes is much better than folic acid absorption itself shown in Table 5. So, these complexes can be used as analogues for folic acid alone.

This may be explained as being due to the high solubility differences between folic acid which is considered as an insoluble material, and its copper and iron complexes are shown in Table 1. 

## 4. Conclusion

The complexation between transition metal ions as (Fe(III) and Cu (II)) with folic acid resulted in the formation (1 : 2) molar ratio (metal : folic acid). Folic acid acts as a bidentate ligand via two monodentate carboxylate groups giving the general formula: [M(FO)_2_ (H_2_O)_*n*_] · *x*H_2_O, where FO = folate anion; *n * = 2 and *x* = 2 or 3. The resulted folate compounds were assigned by infrared, optical measurements. Thermogravimetric analysis and atomic absorption of folate complexes. The transition metals folic acid complexes are more preferred than folic acid itself as a drug in the human body because the absorption efficiencies of the transition metal complexes are higher than those the folic acid.

## Figures and Tables

**Figure 3:**
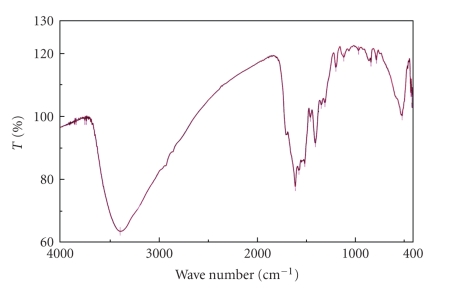
I.R. Spectrum of iron folate complex.

**Table 1:** Elemental analysis and solubility of the complexes.

Metal complexes	Solubility (g/L)	Metal		C		H		N	
		Cal %	Found %	Cal %	Found %	Cal %	Found %	Cal %	Found %
K_2_[Cu(FO)_2_(H_2_O)_2_]2H_2_O(yellowish green)	2.18 g/L	5.83	6.09	41.85	41.08	3.67	3.27	17.99	16.30
K[Fe(FO)_2_(H_2_O)_2_]3H_2_O(dark brown)	1.67 g/L	5.47	4.87	42.98	42.29	3.83	3.36	18.47	17.91

**Table 2:** IR frequencies (Cm^1^ ) of folic acid (FO) and its metal complexes.

Assignments	Compounds		
	Folic acid	Fe-folate complex	Cu-folate complex
*ν*(OH); H_2_O	3415	3381	3388
	3415		
*ν*(NH) *amide *	3230	3175	3220
*ν*_as_(CH)	3106	3090	3030
	2926	2930	2930
*ν*_s_(CH)	2840	2820	2830
		2770	2805
*ν*(C=O) *amide I *	1652	1658	1655
*ν*(COOH)	1718	—	—
*ν*_as_(COO−)	1569	1613	1603
*δ*(CH)	1483	1448	1445
	1413	1401	1400
*ν*_s_(COO−)	1453	1369	1350
*δ*(NH) *amide II *	1528	1520	1532
*ν*_as_(CC)	1323	1303	1300
*ν*(CN)	1230	1185	1186
	1191	1106	1102
	1106		
*ν*_s_(CC)	971	980	955
*δ*(CC)	764	767	766
*ν*(M–O)	—	587	585
		520	521

**Table 3:** Asymmetric and Symmetric Stretching Vibrations of the Carboxylate group.

Compound	*ν*_as_(COO)	*ν*_s_(COO)	*ν* Δ = *ν*_as_(COO^−^) − *ν*_s_(COO^−^)	Bonding mode
Folic acid	1569	1453	116	—
K[Fe(FO)_2_((H_2_O)_2_] · 2H_2_O	1631	1369	262	Mono dentate
K_2_[Cu(FO)_2_(H_2_O)_2_] · 2H_2_O	1603	1350	253	Mono dentate

**Table 4:** The concentration of Cu and Fe elements in mice blood serum in case of folic acid and folic acid metal complex.

Elements	Cu (mg/L) (after inject of folic acid)	Cu (mg/L) (after inject complex)	Fe (mg/L) (after inject of folic acid)	Fe (mg/L) (after inject complex)
Mice 1	5.1	6.4	7.9	12.4
Mice 2	4.8	6.9	8.3	13.2
Mice 3	4.9	7.6	7.7	11.8
Mice 4	5.1	7.2	9.2	14.7
Mice 5	5.1	6.9	10.2	16.9

**Table 5:** Comparison between the normal, folic acid case and folic acid metal complex case percentage of Cu and Fe elements in the serum of mice.

Metal ion	Normal concentration in the blood mg/L	After inject of folic acid mg/L	After inject complex mg/L
Cu (mg/L)	1.18	5.00 ± 0.21	7 ± 0.6
Fe (mg/L)	2.1	9.2 ± 0.15	13.8 ± 0.8
